# Supplementary material for: Synergistic Effect of TiO2-Nanoparticles and Plant Growth-Promoting Microorganisms on the Physiological Parameters and Antioxidant Responses of Capsicum annum Cultivars
Source: Antioxidants (Basel). 2025 Jun 10;14(6):707. doi: 10.3390/antiox14060707 (PMC12189501; doi:10.3390/antiox14060707)
Supplement: Supplementary file 1 [file antioxidants-14-00707-s001.zip › antioxidants-3642509-supplementary.pdf]

Article

Synergistic Effect of TiO_2-Nanoparticles and Plant Growth-Promoting Microorganisms on the Physiological Parameters and Antioxidant Responses of *Capsicum annum* Cultivars

Atiya Bhatti [1], Araceli Sanchez-Martinez [2], Gildardo Sanchez-Ante [1], Daniel A. Jacobo-Velázquez [3], Joaquín Alejandro Qui-Zapata [4], Soheil S. Mahmoud [5], Ghulam Mustafa Channa [1], Luis Marcelo Lozano [1], Jorge L. Mejía-Méndez [6,*], Edgar R. López-Mena [1,*] and Diego E. Navarro-López [1,*]

[1] Tecnologico de Monterrey, Escuela de Ingeniería y Ciencias, Ave. General Ramon Corona 2514, Zapopan 45138, Jalisco, Mexico; atiya.bhatti10@gmail.com (A.B.); gildardo.sanchez@tec.mx (G.S.-A.); gmchanna139@gmail.com (G.M.C.); marcelo.lozano@tec.mx (L.M.L.)

[2] Departamento de Ingeniería de Proyectos, CUCEI, Universidad de Guadalajara, Av. José Guadalupe Zuno # 48, Industrial los Belenes, Zapopan 45157, Jalisco, Mexico; araceli.sanchez46@academicos.udg.mx

[3] Tecnologico de Monterrey, Institute for Obesity Research, Ave. General Ramon Corona 2514, Zapopan 45201, Jalisco, Mexico; djacobov@tec.mx

[4] Biotecnología Vegetal, Centro de Investigación y Asistencia en Tecnología y Diseño del Estado de Jalisco A.C., Camino Arenero 1227, El Bajío, Zapopan 45019, Jalisco, Mexico; jqui@ciatej.mx

[5] Department of Biology, The University of British Columbia, Okanagan Campus, 1177 Research Road, Kelowna BC V1V 1V7, Canada; soheil.mahmoud@ubc.ca

[6] Programa de Edafología, Colegio de Postgraduados, Campus Montecillo, Carretera México Texcoco km 36.4, Montecillo 56264, Mexico

* Correspondence: mejia.jorge@colpos.mx (J.L.M.-M.); edgarl@tec.mx (E.R.L.-M.); diegonl@tec.mx (D.E.N.-L.)

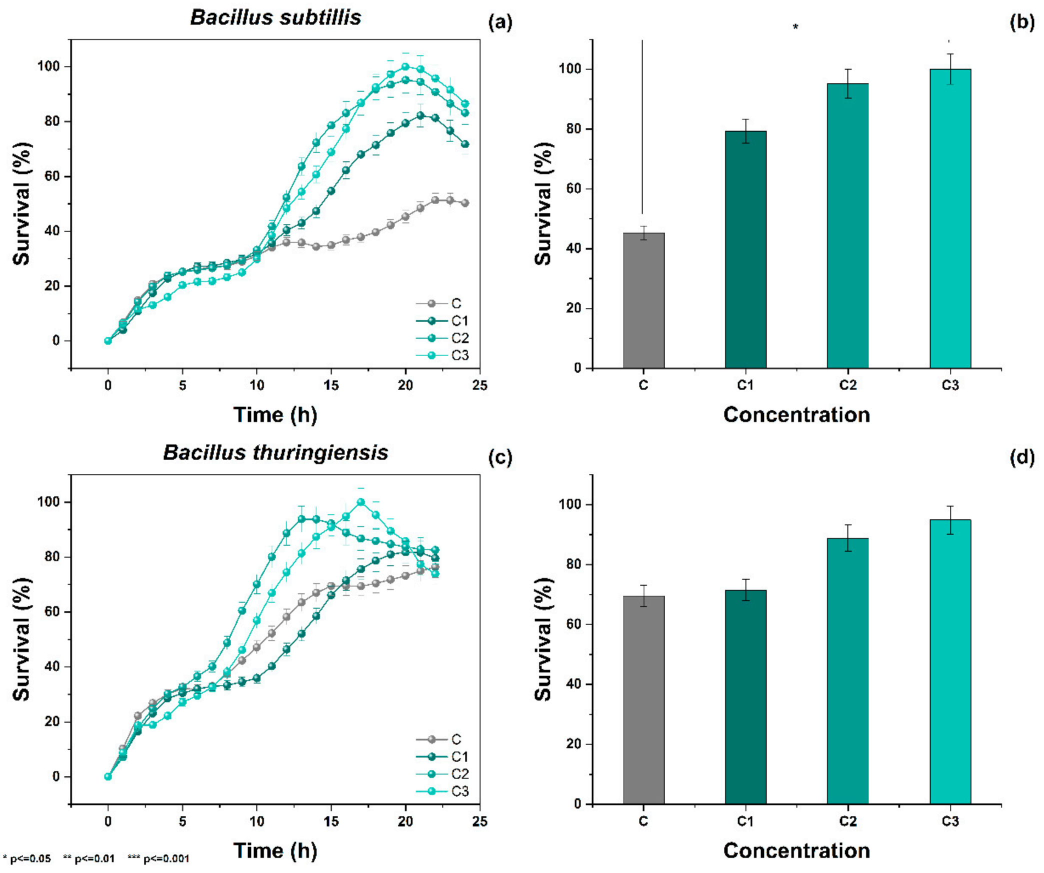

Figure S1. Effect of TiO_2-NPs in kinetic growth of *B. subtilis* and *B. thuringiensis*. C1 (50 µg/mL), C2 (100 µg/mL), and C3 (150 µg/mL) are associated with the tested concentrations. (a) survival percentage during *B. subtilis* growth kinetics, (b) survival percentage at 24 hours of *B. subtilis* growth, (c) survival percentage during *B. thuringiensis* growth kinetics, (d) survival percentage at 24 hours of *B. thuringiensis* growth. The data are represented as the mean ± standard deviation (SD) from three independent experiments, with the resulting analysis of variance (ANOVA) yielding a p-value of less than 0.05.

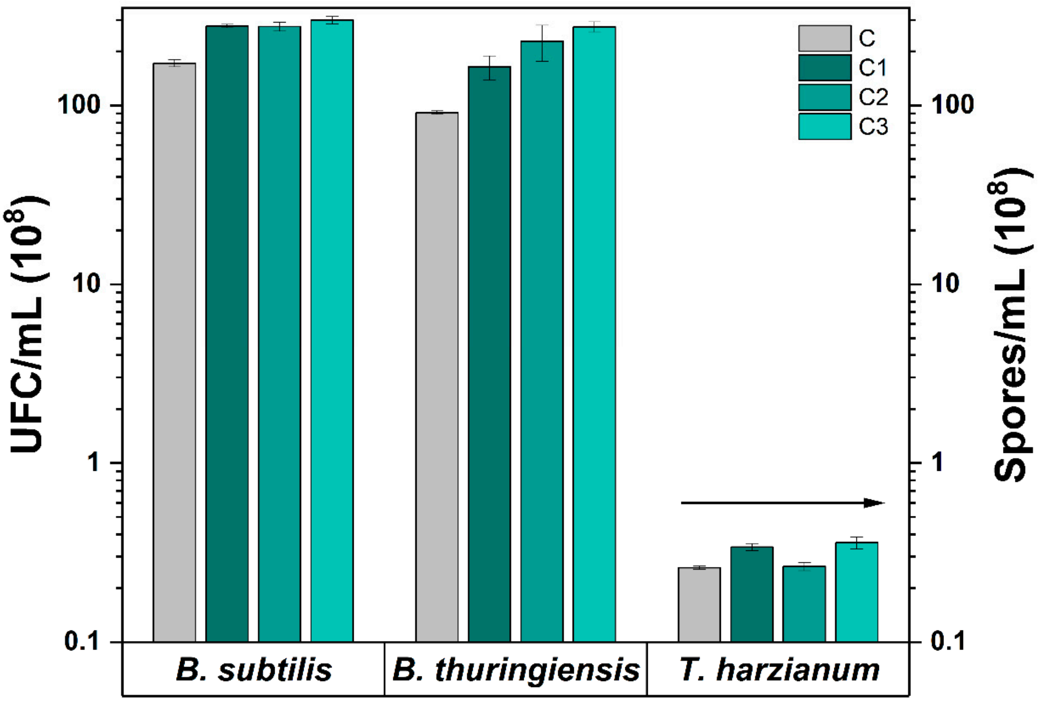

Figure S2. Effect of TiO_2-NPs in the CFUs of *B. subtilis* and *B. thuringiensis*, and spore production of *T. harzianum*. C1 (50 µg/mL), C2 (100 µg/mL), and C3 (150 µg/mL) are associated with the tested concentrations. The data are represented as the mean ± standard deviation (SD) from three independent experiments, with the resulting analysis of variance (ANOVA) yielding a p-value of less than 0.05.

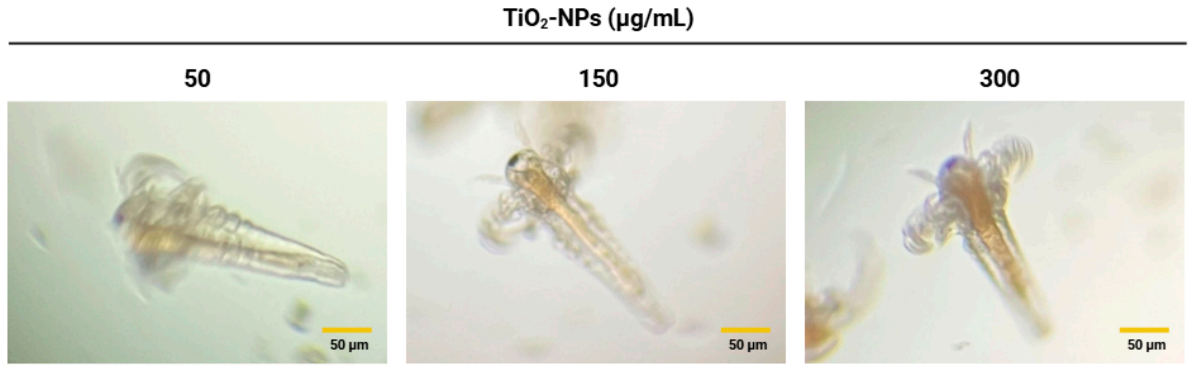

Figure S3. Toxicity evaluation of TiO_2-NPs in *A. salina* nauplii.
